# A Corner on Prostitution

**Published:** 1913-02

**Authors:** 


### A Corner on Prostitution

The business of prostitution in New York City has amalgamated itself into a trust for the purpose of systematically paying tribute to the police officials, in defiance of law, that they may not be molested in their business. According to *Current Opinion,* there are now 35,000 disorderly women in that city, who are thus forced into this trust, and the average price they pay for this protection is from $60 to $1000 per month. Why not Congress regulate this trust as well as some others it has attempted of recent date?

Prostitution is at the foundation of our national decadence and should be eradicated. There are 300,000 registered prostitutes in the United States and 2,000,000 who ply their trade clandestinely. It costs the American people over $3,000,000,000 annually for illicit venery—$1,000,000,000 more than the cost of intoxicating liquors.

